# Risk assessment of arrhythmias related to three antiseizure medications: a systematic review and single-arm meta-analysis

**DOI:** 10.3389/fneur.2024.1295368

**Published:** 2024-02-14

**Authors:** Yulong Li, Shen Su, Mengwen Zhang, Limin Yu, Xinyuan Miao, Hongjun Li, Yanping Sun

**Affiliations:** ^1^Department of Neurology, The Affiliated Hospital of Qingdao University, Qingdao, China; ^2^Department of Gastroenterology, The Affiliated Hospital of Qingdao University, Qingdao, China; ^3^Department of Neurology, Tai’an City Central Hospital, Tai’an, China

**Keywords:** antiseizure medications, epilepsy, arrhythmias, levetiracetam, lacosamide, perampanel

## Abstract

**Objective:**

Antiseizure medications (ASMs) are first line therapy for seizure disorders. Their effects on arrhythmias, especially the risk of arrhythmias associated with lacosamide (LCM), levetiracetam (LEV), and perampanel (PER), have been intensely investigated.

**Methods:**

We searched four databases (PubMed, EMBASE, Cochrane Library, and Web of Science) until August 6, 2023. We used a common effects model and reported data as pooled incidence with 95% CIs. Meta-analyses were conducted to elucidate the risk of arrhythmias with different drugs, and Egger’s regression was performed to detect publication bias analysis.

**Results:**

We included 11 clinical trials with 1,031 participants. The pooled incidence of arrhythmias in the LEV group was 0.005 (95% CI: 0.001-0.013), while it was 0.014 in the LCM group (95% CI: 0.003-0.030). Publication bias analyses indicated no significant bias in the LEV group (*t* = 0.02, df = 4, *p*-value = 0.9852) but a significant bias in the LCM group (*t* = 5.94, df = 3, *p*-value = 0.0095). We corrected for this bias in the LCM group using the trim-and-fill method, which yielded a similar pooled incidence of 0.0137 (95% CI: 0.0036-0.0280), indicating good reliability. Due to insufficient studies, we could not conduct a meta-analysis for PER, and we analyzed them in our systematic review.

**Conclusion:**

The use of LCM significantly elevated the risk of arrhythmias, while LEV had non-significant arrhythmogenic effects. As for the arrhythmogenic effects of PER, more clinical trials are needed in the future.

## 1 Introduction

Antiseizure medications (ASMs) are the first line treatment for seizure disorders (1, 2). There is a growing body of evidence that some ASM’s are associated with an increased risk for cardiac arrythmias (3–6). We performed a systematic review to determine the relative risk of arrythmias on three common newer generation ASMs: lacosamide (LCM), levetiracetam (LEV), and perampanel (PER). It has been reported that about 80% of patients experience symptom relief when taking medication, and approximately 50% of those who undergo medication withdrawal are successful in preventing recurrence of epileptic seizures (7). However, despite the effectiveness of ASMs in controlling seizures and reducing their frequency and severity (8), approximately one-third of patients still experience recurrent seizures. It is important to note that ASM is a double-edged sword, as it can cause adverse reactions such as arrhythmia, which is often observed in patients with epilepsy (4, 9). Both epilepsy and ASMs were found to be associated with an elevated risk of cardiovascular diseases. ASMs prolong the QT interval by closing ion channels or delaying their opening, thereby affecting cardiac rhythm and increasing the risk of arrhythmias in susceptible individuals, which provides a pathophysiological basis for ASM-induced arrhythmias (10–12).

The drug interactions and adverse effects of ASMs are great challenges for the quality of life of people with epilepsy (13). Cardiac arrhythmias, in particular, can directly affect the lives of patients with epilepsy.

We chose three newer and more commonly used ASMs, namely, LEV, LCM, and PER. As a newer ASM, LEV is emerging as a versatile drug compared to metformin and aspirin. It has been used for the treatment of epilepsy, pain, ulcerative colitis, and Parkinson's disease, as well as cognitive and psychiatric disorders, and therefore its side effects deserve our attention (14–17). LCM enhances slow sodium channel inactivation in both the brain and heart, which reduces the channel availability over a long period, particularly during epileptic seizures, and reduces interictal discharges (18). LCM-related arrhythmias have also been investigated (19). PER has often been used as an adjunctive drug in epilepsy. Since the FDA approved it for use as a single agent in treating epilepsy, its monotherapy has become a hot research topic (20).

The purpose of this study is to preliminarily evaluate the incidence of cardiac arrhythmia with the use of three ASMs (LCM, LEV, and PER), and provide guidance for the treatment and management of epilepsy.

The meta-analysis is registered in PROSPERO, registration number CRD42023458029 (Supplementary file 3). It was conducted following the Preferred Reporting Items for Systematic Reviews and Meta-Analyses (PRISMA) statement (Figure 1).

## 2 Materials and methods

**Figure 1:**
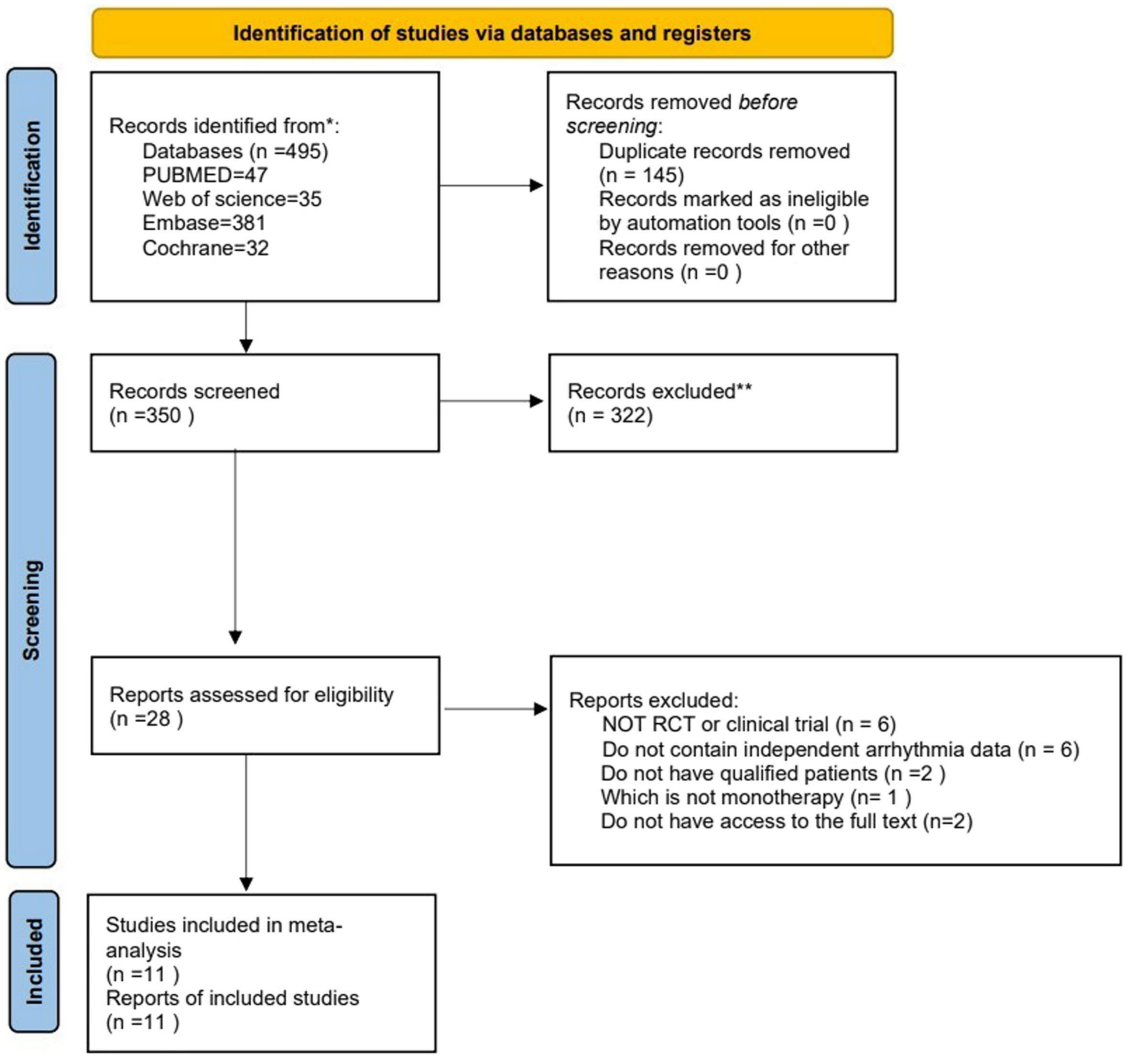
Literature search process and study selection profile. We obtained 495 articles through a search across four databases; following screening, we ultimately selected 11 pertinent publications.

### 2.1 Inclusion and exclusion criteria

#### 2.1.1 Inclusion criteria

1. Study: Randomized controlled trials (RCTs) or clinical trials
2. Participants: Patients with epilepsy
3. Interventions: Patients with epilepsy who were treated with LEV, PER, or LCM as a monotherapy
4. Outcomes: The outcomes including any type of arrhythmias or unclassified arrhythmias.

#### 2.1.2 Exclusion criteria

1. Treatment of diseases other than epilepsy
2. The detailed data on efficacy and safety profiles were not available
3. Patients have other diseases that affect arrhythmias
4. Pregnant women with epilepsy
5. No adverse reactions related to arrhythmia were found or ECG monitoring was not mentioned in the safety analysis.

### 2.2 Search strategy and study selection

We searched PubMed, Cochrane Library, EMBASE, and Web of Science databases up to August 6, 2023. We utilized both subject headings and free text terms in our search strategy to ensure a comprehensive search. Our search terms included “ASM”, perampanel/PER”, “levetiracetam/LEV”, “lacosamide/LCM”, “arrhythmias”, and “monotherapy”. In the search for the articles on PER, we added "monotherapy" into our search strategy, since many studies used PER as adjuvant therapy. The detailed search strategy is described in Supplementary file 2. Additionally, references included in eligible research and reviews were checked to see whether any additional studies met our eligibility requirements.

Two independent investigators (Yulong Li and Shen Su) searched the databases and screened the articles according to the inclusion and exclusion criteria. Any disagreement were resolved through discussion with a third investigator.

### 2.3 Data extraction and quality assessment

We extracted the following data into an Excel spreadsheet from each study with a predefined form consisting of the author, publication year, country, type of study, number of arrhythmias, types of arrhythmias, number of patients, mean/median age, female proportion, doses used for LEV, PER, and LCM, and mode of administration.

The Cochrane risk of bias tool, RoB 2, was used to evaluate the quality of the RCTs. It includes six items: randomization process, deviations from intended interventions, missing outcome data, measurement of the outcome, selection of the reported result, and overall bias. The risk of bias in each item was rated as low, some concerns, or high.

### 2.4 Data synthesis and analysis

After extracting the data, we first calculated the proportions. Then, we tested the normality of our data using the Shapiro–Wilk test. If they were normally distributed, we directly used the proportions as the effect sizes. However, if the distribution was skewed, they would be transformed to approximate a normal distribution using the most suitable one from the following transformation methods: logit transformation; arcsine transformation; Freeman–Tukey double arcsine transformation, and log transformation.

We used a meta-analytical approach with data synthesis techniques to investigate associations of arrhythmias incidence with the use of LEV and LCM. We estimated the pooled incidence rates and their corresponding 95% confidence intervals (95% CIs). Heterogeneity between the included studies was assessed using the *I*^2^ test. *I*^2^ > 50% was considered indicative of significant heterogeneity. We conducted meta-analyses using the Mantel–Haenszel method. A random effects model was used if *I*^2^ ≥ 50%, and a common effects model was used if *I*^2^ < 50% and *p* >0.05. The results of our meta-analyses were presented as pooled incidences and their 95% CIs, as shown in the forest plots. A *p*-value of < 0.05 was considered statistically significant.

If there was a significant heterogeneity across studies, subgroup analyses or funnel plots would be conducted to further explore the source of heterogeneity. All the above statistical analyses were performed using R 4.2.3.

## 3 Results

The initial database search yielded a total of 495 articles. After removing 145 duplicate records, 350 articles were eligible. After screening the titles and abstracts, 322 articles were excluded. After the full-text screening, 17 articles were excluded. Finally, 11 RCTs or clinical trials were included in this meta-analysis. The PRISMA flowchart (Figure 1) shows the study selection process.

### 3.1 Study and participant characteristics

The detailed demographics and study characteristics are shown in Supplementary Table S1. A total of 11 articles with 1,031 epileptic patients were included in our meta-analyses, including six articles on LEV (21–26) and five articles on LCM (27–31). Among these 11 studies, there were six RCTs and five clinical trials, according to the type of study. However, all the included studies conducted safety analyses, with one or more types of cardiac arrhythmias as outcomes.

The articles on PER were not sufficient enough for us to carry out a meta-analysis. The qualitative description of previous articles on PER is presented in the “Discussion” section.

### 3.2 Quality assessment of the included studies

**Figure 2:**
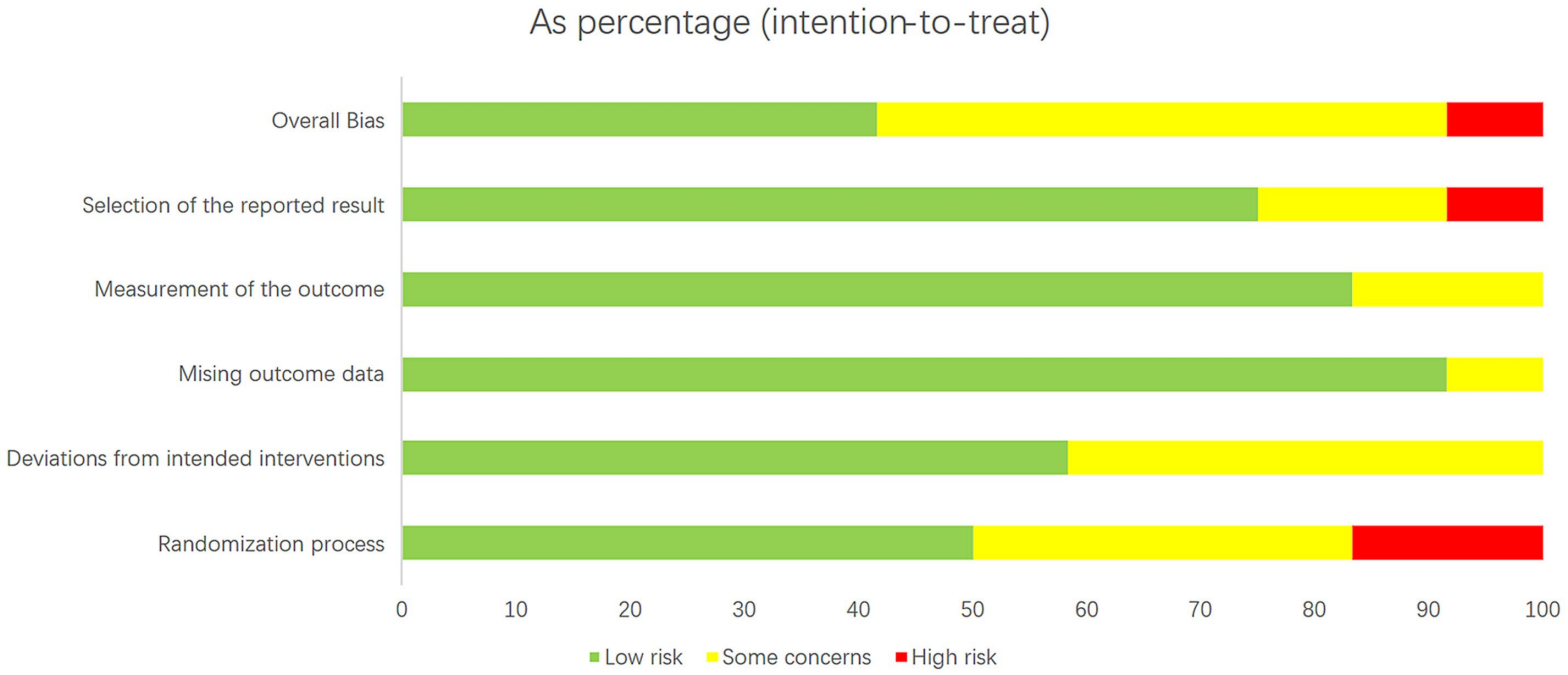
Literature quality evaluation results. The RoB 2 tool produces a risk-quality rating scale, with low risk accounting for about 70 percent of the total and high risk for about 10 percent.

**Figure 3:**
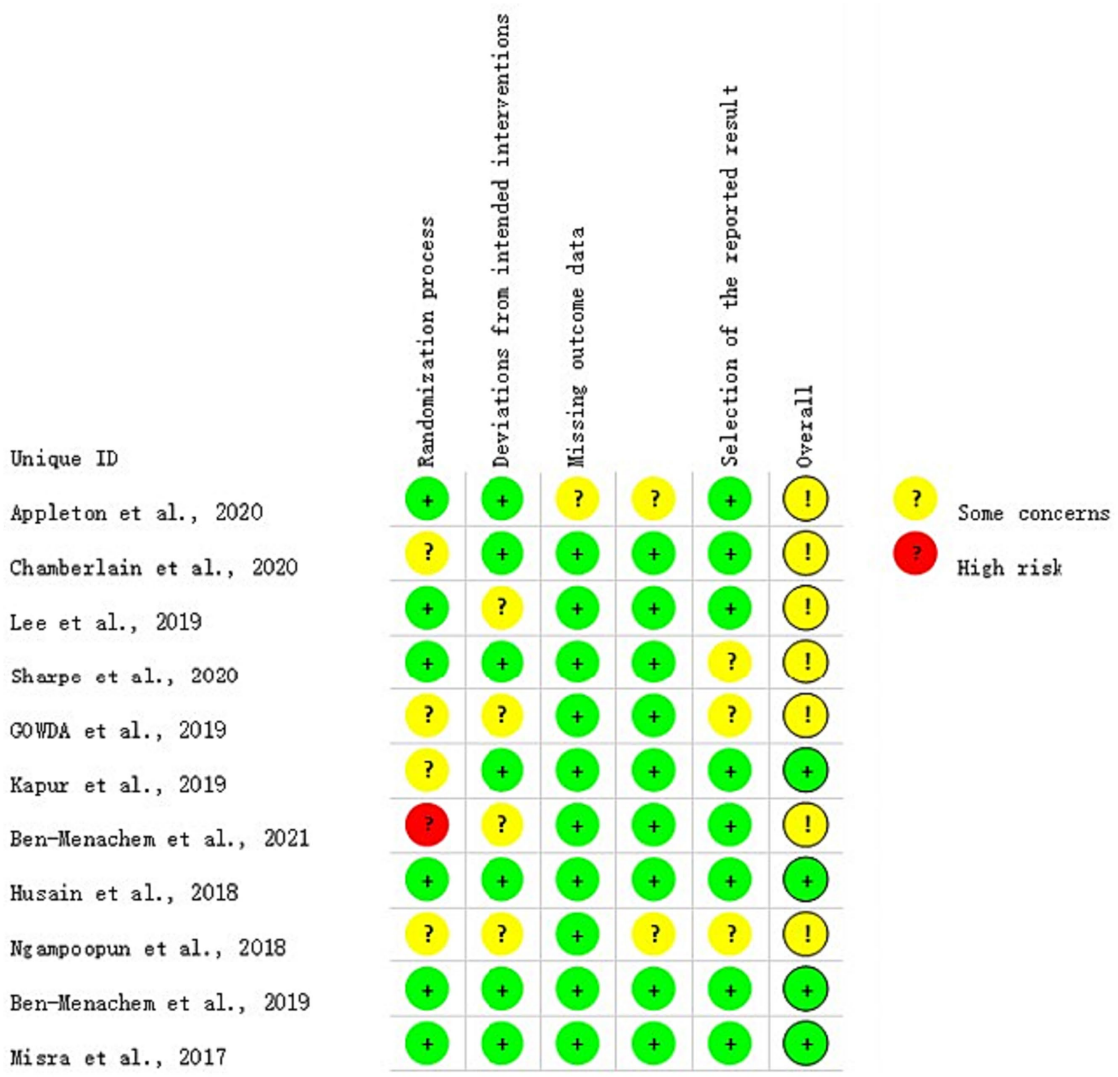
Literature quality evaluation results. Street light map generated simultaneously by the RoB 2 tool, containing only one high-risk “light”.

The included studies were assessed for study quality using RoB 2. Among the 11 included articles, 10 did not exhibit a high-risk of bias, indicating the overall good quality of the included studies. All studies demonstrated baseline comparability. However, it should be noted that some studies were clinical trials and blinding could not be fully implemented. The results of the quality assessment are shown in Figures 2, 3.

### 3.3 Efficacy outcomes

#### 3.3.1 Meta-analyses

The 11 included articles studied a total of 1,031 epileptic patients, including 567 patients who had LEV and 464 patients who had LCM. As for the outcomes, there were 4 cases with arrhythmias in the LEV group and 11 cases in the LCM group. The incidences of arrhythmias were 0.71% and 2.37% for the LEV and LCM groups, respectively.

**Figure 4:**
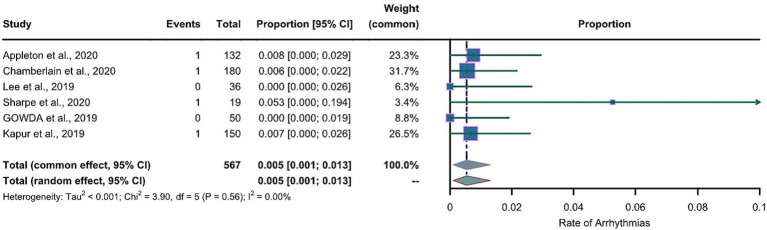
Forest plot of arrhythmia incidence in the LEV group. The pooled incidence of arrhythmias in LEV group is 0.005 (0.001-0.013) with the common effect model.

**Figure 5:**
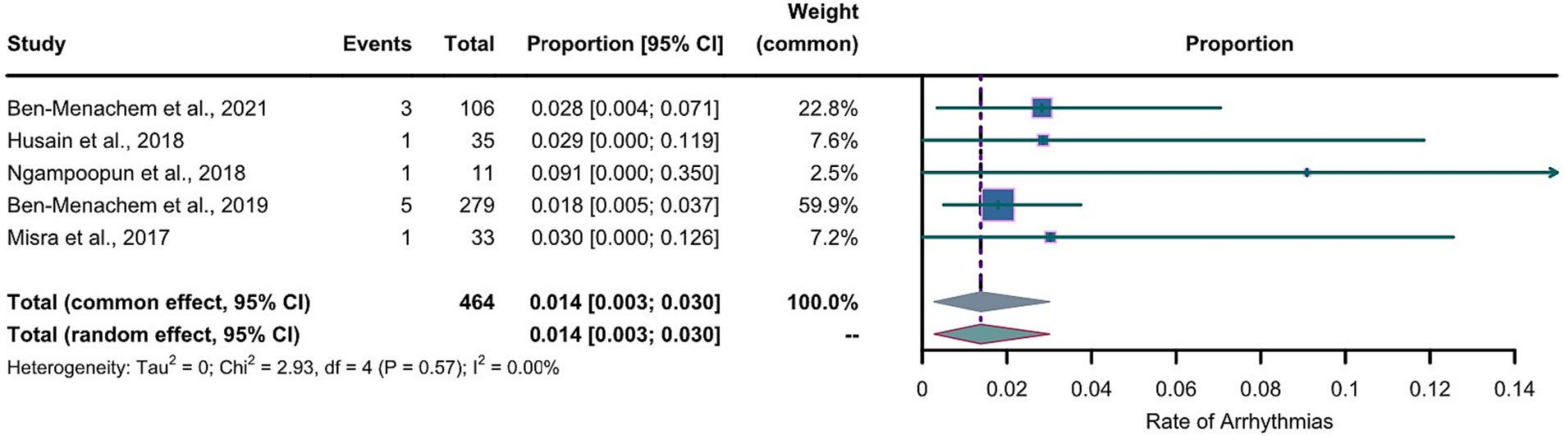
Forest plot of arrhythmia incidence in the LCM group. The pooled incidence of arrhythmias in LCM group is 0.014 (0.003-0.030) with the common effect model.

For the LEV group, the arcsine transformation (*p*=0.155) best fitted a normal distribution among the transformation methods when calculating the effect size. For the LCM group, the Freeman-Tukey dual arcsine transformation best fitted a normal distribution (*p*=0.1634). The heterogeneities in both groups were not statistically significant (LEV group: *I*^2^=0, p=0.56; LCM group: *I*^2^=0, *p*=0.57). We collected the safety profiles from the included articles and meta-analyzed these data. Notably, due to the observed substantial heterogeneities in both LEV and LCM groups, we employed a common-effects model, which inherently accounts for both within-study and between-study variances. The results of our common-effects model showed that the pooled incidence rates of cardiac arrhythmias were 0.005 in the LEV group (95%CI 0.001; 0.013) and 0.014 in the LCM group (95%CI 0.003; 0.030), as illustrated in two forest plots (Figures 4, 5).

Figures 4, 5 reveal extreme values; therefore, we removed one article from the LEV group and one article from the LCM group which were the sources of these extreme values. After the exclusion, we further conducted meta-analyses separately for the LEV and LCM groups, as shown in supplementary material (Supplementary Figures S1, S2). The results of the meta-analyses showed that after the exclusion, pooled incidences of arrhythmias were 0.005 in the LEV group (0.001-0.012), and 0.018 in LCM group (0.006-0.034), which showed slight differences with the results before the exclusion.

#### 3.3.2 Subgroup analysis

Subgroup analyses by dosage were conducted in both groups. Based on the dosage of LEV, participants in the LEV group were divided in the low-dose LEV group (less than 60mg/kg) and the high-dose LEV group (equal to 60mg/kg). We conducted meta-analyses on the subgroups. The pooled incidence rate of arrhythmias in the low-dose LEV group was 0.004 (95% CI 0.000-0.017) and the rate in the high-dose LEV group was 0.006 (95% CI 0.001-0.017), both of which were similar to the previous pooled incidence of 0.005 in the primary meta-analysis. It can be inferred that changes in dosage do not affect the risk of arrhythmias associated with LEV (Supplementary Figure S3).

In the LCM group, participants were divided into two subgroups based on the dosage: one group received a fixed dosage of LCM, while the other group received three stepwise dose increases until effective seizure control was achieved. Using a random-effects model, the pooled incidence of arrhythmias in the fixed dosage group (0.031, 95% CI 0.000-0.091) was higher than that in the stepwise dose increase group (0.020, 95% CI 0.007-0.037), indicating that dosage might be a factor influencing the risk of arrhythmias associated with LCM (Supplementary Figure S4).

#### 3.3.3 Publication bias

We conducted Egger’s regression method to test the publication bias in both the LEV and LCM groups, showing no publication bias in the LEV group (*t* = 0.02, df = 4, *p*-value = 0.9852) and a substantial publication bias in the LCM group (*t* = 5.94, df = 3, *p*-value = 0.0095). We used the trim-and-fill method to adjust for the publication bias and produced a new funnel plot (Supplementary Figure S5). After the adjustment, we obtained a pooled incidence of 0.0137 (95% CI 0.0036-0.0280), which was only slightly different from our initial result of 0.0140, indicating a publication bias within an acceptable range.

## 4 Discussion

The primary findings of our study indicate the pooled incidence of cardiac arrythmias for LCM was 1.4% (*I*^2^=0, *p*=0.57) versus 0.5% for LEV (*I*^2^=0, *p*=0.56), which is important and provides evidence for clinicians to weigh the relative risk of cardiac arrhythmias in two commonly used ASMs. It also supplements the research on drug induced arrhythmias.

In 2021, the World Health Organization initiated a resolution on epilepsy and other neurological disorders and called for improving the prevention and diagnosis of neurological disorders (including epilepsy) as well as the treatment and rehabilitation of patients (32). Although they are frontline treatment for epilepsy, ASMs have two major drawbacks, including adverse reactions and drug resistance (33).

As a representative ASM, LCM blocks sodium channels and enhances their slow inactivation (34–36). Previous studies did not show a significant effect of LCM on cardiac safety, with the exception of prolonging the PR interval rather than the QR interval (37, 38). According to drug developer records obtained from the FDA, LCM can cause atrioventricular block and ventricular tachycardia. Therefore, LCM should be used with caution in patients with other contributing factors for arrhythmia, including pre-existing cardiovascular conduction disease, medications that affect cardiac conduction system, and diabetic neuropathy (19). Recent studies reported that ventricular tachycardia (29.4%) was the most commonly observed LCM-related arrhythmia, followed by new-onset atrial fibrillation (17.6%), complete heart block (17.6%), Mobitz type 1 atrioventricular block (11.8%), sinus pauses (11.8%), pulseless electrical activity (5.9%), and QRS complex widening (5.9%). As mentioned above, conduction block is a common adverse effect of LCM, following ventricular tachycardia and atrial fibrillation. Traditional sodium channel blocking agents, such as carbamazepine and phenytoin sodium, might have synergistic effects with LCM, and therefore LCM should be used with caution when used with other drugs (11, 39).

Compared to LCM, LEV has better safety and efficacy although it may have cardiac toxicity (40). A case report found that the pharmacokinetics of LEV in overdose appeared to be similar to therapeutic LEV dosing after analyzing a case of LEV poisoning (41). Two previously RCTs used healthy subjects who took LEV as the intervention group, with healthy subjects who had LEV as reference. The two groups showed no significant differences in QT interval, PR interval, Tpe/QT ratio, and Tp-e/QTc ratio (42, 43). However, in some case reports, patients with pre-existing heart disease experienced worsened cardiovascular conditions after taking LEV treatment, suggesting the cardiac effects of LEV (44, 45). Another common ASM is PER which has been used as both adjuvant therapy and monotherapy (46). PER is a selective non-competitive AMPA receptor antagonist that works by reducing excessive glutamate-mediated neurotransmission to control epileptic seizures, and it is a potential broad-spectrum ASM (35, 47). However, a previous study showed that PER did not exert effects on cardiac repolarization and did not prolong the QT interval in healthy participants after taking PER for seven days (48). What is more, a very recent study also suggested that PER could reduce the risk of arrhythmias by activating the parasympathetic nerves (49). Although arrhythmia-related adverse reactions of PER are not common, possible long-term adverse effects of PER remain to be uncovered.

Our meta-analyses showed that in patients with epilepsy, the pooled incidences of arrhythmias were 1.4% and 0.5% for the LCM and LEV group, respectively. The Council for International Organizations of Medical Sciences (CIOMS) recommends that the frequency of adverse reactions is expressed as very common (>=1/10), common or frequent (>=1/100 and <1/10), uncommon or infrequent (>=1/1000 and <1/100), rare (>=1/10000 and <1/1000), and very rare (<1/10000) (50). According to the CIOMS criteria, LCM can be rated as "frequent" while LEV can be rated as "infrequent". Since our articles on PER were insufficient, we could not rate PER based on the CIOMS criteria. This may imply that when a novel drug is introduced into the market, there are insufficient eligible studies on it and some of its long-term adverse effects remain undiscovered.

Both LEV and LCM are the first-line treatment options for focal epilepsy (1). LCM selectively acts on slow sodium channel inactivation and prolongs sodium channel inactivation, thereby reducing the excitability of neurons. LEV binds to the unique synaptic vesicle protein 2A (SV2A) to decrease the rate of vesicular release, thereby reducing the release of the neurotransmitter GABA (51). A study on the safety of LCM and LEV reported that LCM was more likely to induce arrhythmias than LEV (52), which was consistent with our conclusion that LEV was safer than LCM for arrhythmia treatment. LEV was the first choice for adjunctive treatment of refractory epilepsy. PER and LCM showed no advantage in efficacy and safety than LEV (53). Since the psychiatric side effects of LEV and PER are common, individualized medication of LEV and PER is recommended.

As an old ASM, phenytoin sodium has both adverse and protective effects on the heart. One RCT we included used phenytoin sodium in the control group, and the incidence of arrhythmia related to phenytoin sodium was 0.023, which was higher than our pooled incidences in LEV and LCM groups from our meta-analyses. Intravenous phenytoin sodium has severe adverse effects including severe arrhythmias, skin reactions, ventricular fibrillation, and even death (54). Phenytoin sodium also can reduce the activity of the cardiac ryanodine receptor 2 to provide cardio protection (55).

Lamotrigine (LTG) is an old ASM which inhibits the release of the excitatory neurotransmitters via blocking voltage-sensitive sodium channels (56). A RCT suggested that it was a better choice for the treatment of focal epilepsy than LEV (57, 58). Compared to LCM, LTG may have additional effects on calcium channels and therefore is more likely to cause arrhythmias. Compared with LEV, LCM, and PER, LTG has more allergic reactions which cause indirect non-pharmacologic arrhythmias, and therefore we did not select it in our study. A study found that LTG at therapeutic doses might be linked to modest, non-dangerous QRS widening (59)(60). Another study showed that a toxic plasma concentration level of LTG was associated with an elevated risk of cardiovascular death in elderly LTG users (61).

Another old ASM, oxcarbazepine (OXC), is a sodium channel blocker that stabilizes hyperexcitable neuronal membranes (62). OXC is not recommended for elderly patients with a history of cardiac conduction abnormalities or ventricular arrhythmias (63). A meta-analysis of LEV and OXC as monotherapy showed that their adverse effects did not differ much, but LEV had better seizure control (64). A study showed that epileptic patients who took carboxamide derivatives (including OXC) had a higher risk of arrhythmia than epileptic patients without medication and those without epilepsy (3). Conversely, in a model of male Sprague-Dawley rats, it has been suggested that OXC might serve as a therapeutic agent for ischemia and reperfusion brain (cerebellar) injury induced by cardiac arrest (65).

However, our study also has some limitations. Firstly, not all of the included studies were RCTs. There were insufficient RCTs focusing on the relationships between drugs and cardiac arrhythmias. Secondly, epilepsy itself may lead to cardiac arrhythmias, which might cause confounding bias (66). Thirdly, we could not conduct a subgroup analysis by age. We did not have the raw data on the age of all the participants, and the criteria for dividing participants in age subgroups were not consistent across the included studies. Finally, our sample size was still relatively small. Therefore, the pooled incidences of arrhythmia might be underestimated.

PER has often been used as adjunctive therapy for epilepsy with excellent effectiveness (67). Since the FDA approved PER for use as monotherapy for focal epilepsy, PER showed favorable retention rates and safety profiles (68). Two meta-analyses of RCTs showed that PER had no arrhythmia-related adverse effects (69, 70). However, the rates of other adverse reactions to PER are not extremely low. Most of these adverse reactions are tolerable and mild, and few are severe or life-threatening. On the contrary, some studies have suggested a cardioprotective effect of PER (49), although the number of studies was limited.

Furthermore, ASMs need to be taken for a long time. Patients with epilepsy should undergo regular electrocardiogram monitoring while using ASM to detect the occurrence of cardiac arrhythmias in advance, which is beneficial and necessary for their long-term survival (3). The occurrence of cardiac arrhythmias in patients with epilepsy cannot be solely attributed to ASMs, as epilepsy itself can affect the patients' cardiac rhythm. Perhaps for inpatients with epilepsy who frequently experience cardiac arrhythmias, adding antiarrhythmic drugs to ensure the safety of their hearts could be considered. Discontinuation of the relevant ASM or dose reduction is the preferred measure (71). Mesylate may be considered as an additional treatment to antiepileptic therapy, particularly for patients experiencing cardiac arrhythmias (72). Alternatively, new ASMs can be developed to achieve high effectiveness and safety (73, 74). Additionally, interactive remote patient monitoring devices may be a better way to detect the occurrence of arrhythmias in patients with epilepsy (75).

## 5 Conclusion

The pooled incidence of LCM-related arrhythmia was approximately 0.014, while the pooled incidence of LEV-related arrhythmia was slightly lower at 0.005, suggesting that cardiac arrhythmia as an adverse reaction of LCM and LEV is worth paying attention to. Clinicians should be alert to the drug-induced arrhythmias of all three ASMs when applying them. By monitoring the electrocardiographic manifestations in patients with epilepsy, medication adjustments can be made to achieve better treatment outcomes.

## Data availability statement

The original contributions presented in the study are included in the article/Supplementary material, further inquiries can be directed to the corresponding author.

## Author contributions

YL: Conceptualization, Data curation, Formal analysis, Investigation, Methodology, Software, Writing – original draft. SS: Formal analysis, Investigation, Writing – review & editing. HL: Project administration, Supervision, Validation, Writing – review & editing. MZ: Writing – review & editing. LY: Project administration, Supervision, Writing – review & editing. XM: Investigation, Software, Writing – review & editing. YS: Conceptualization, Methodology, Funding acquisition, Project administration, Resources, Supervision, Validation, Writing – review & editing.
