# Supplementary material for: Risk assessment of arrhythmias related to three antiseizure medications: a systematic review and single-arm meta-analysis
Source: Front Neurol. 2024 Feb 14;15:1295368. doi: 10.3389/fneur.2024.1295368 (PMC10899418; doi:10.3389/fneur.2024.1295368)
Supplement: Supplementary file 1 [file Data_Sheet_1.DOCX]

Supplementary Material

# Supplementary Data

# Supplementary Figures and Tables

For more information on Supplementary Material and for details on the different file types accepted, please see [here](https://www.frontiersin.org/guidelines/author-guidelines#supplementary-material).

## Supplementary Figures.


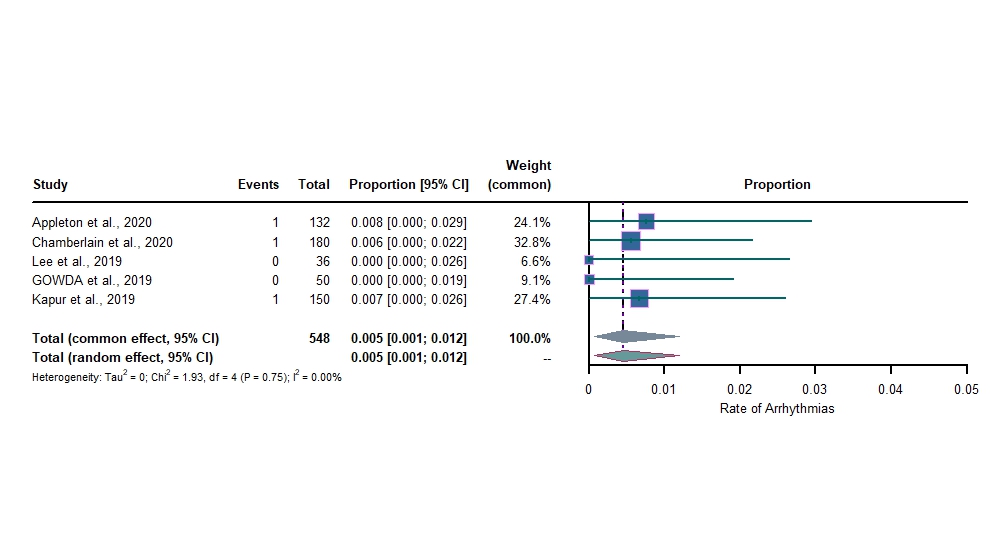


**Supplementary Figure 1.** We removed a piece of literature of LEV group with extreme values, conducted a meta-analysis again, and created a forest plot.


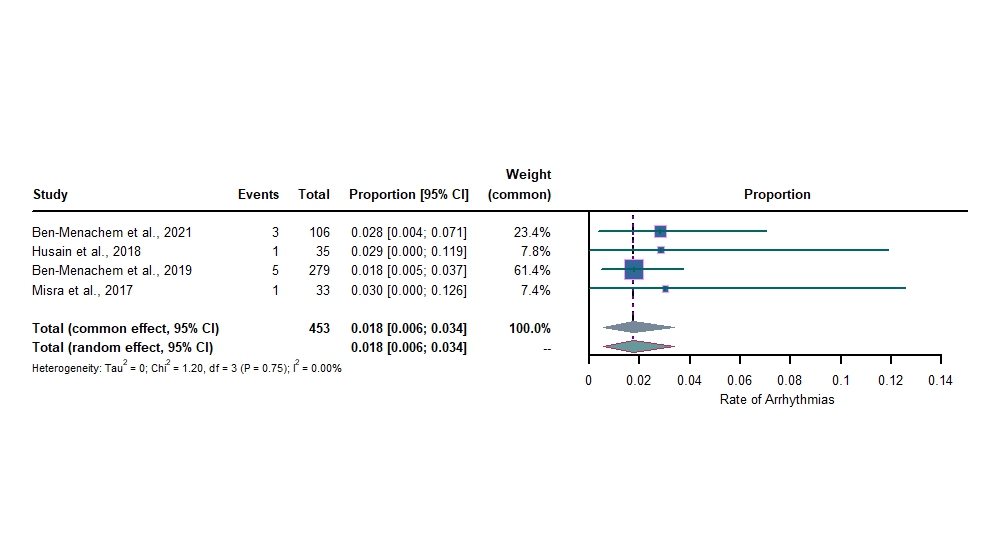


**Supplementary Figure 2.** We removed a piece of literature of LCM group with extreme values, conducted a meta-analysis again, and created a forest plot.


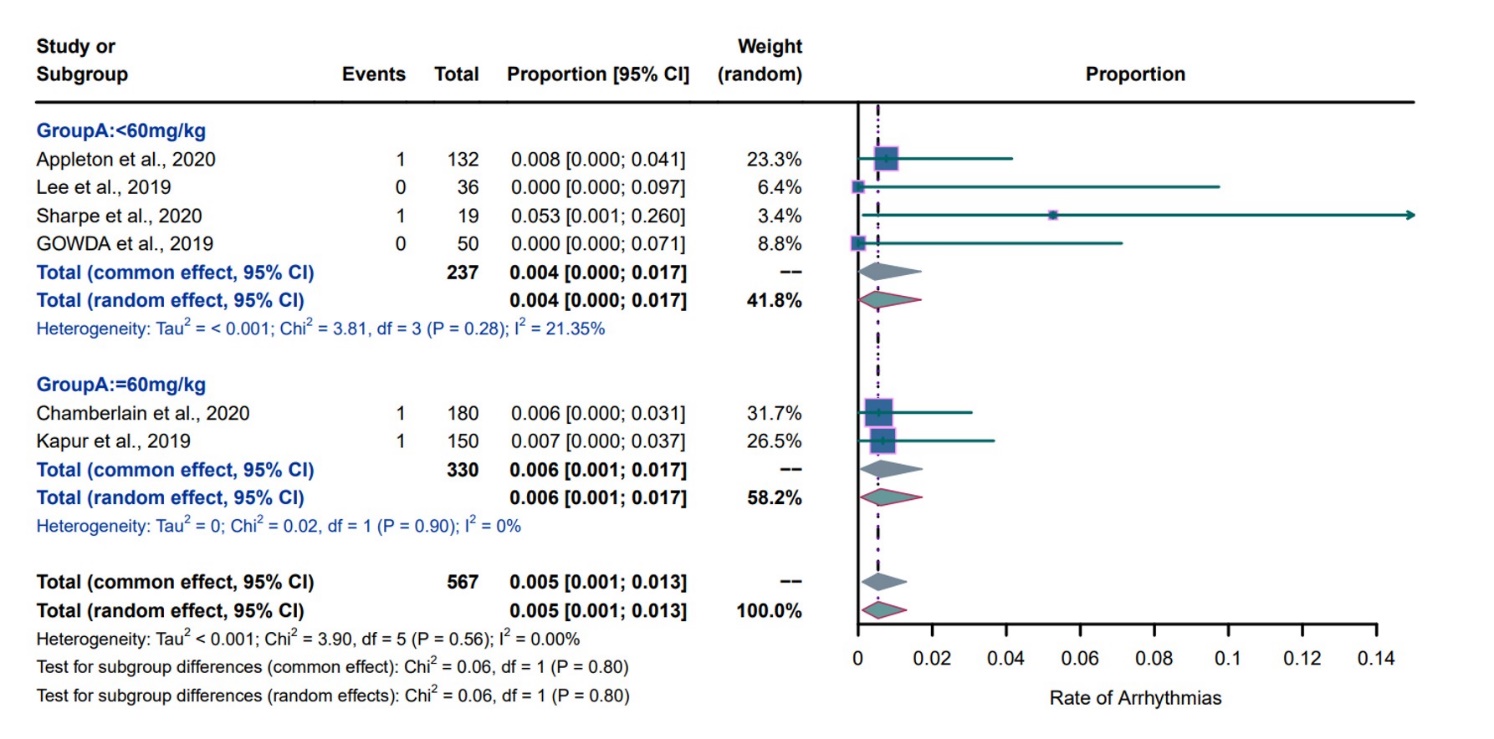


**Supplementary Figure 3.** Subgroup analyses were performed by dividing the LEV group into two subgroups based on the administered dose.

**
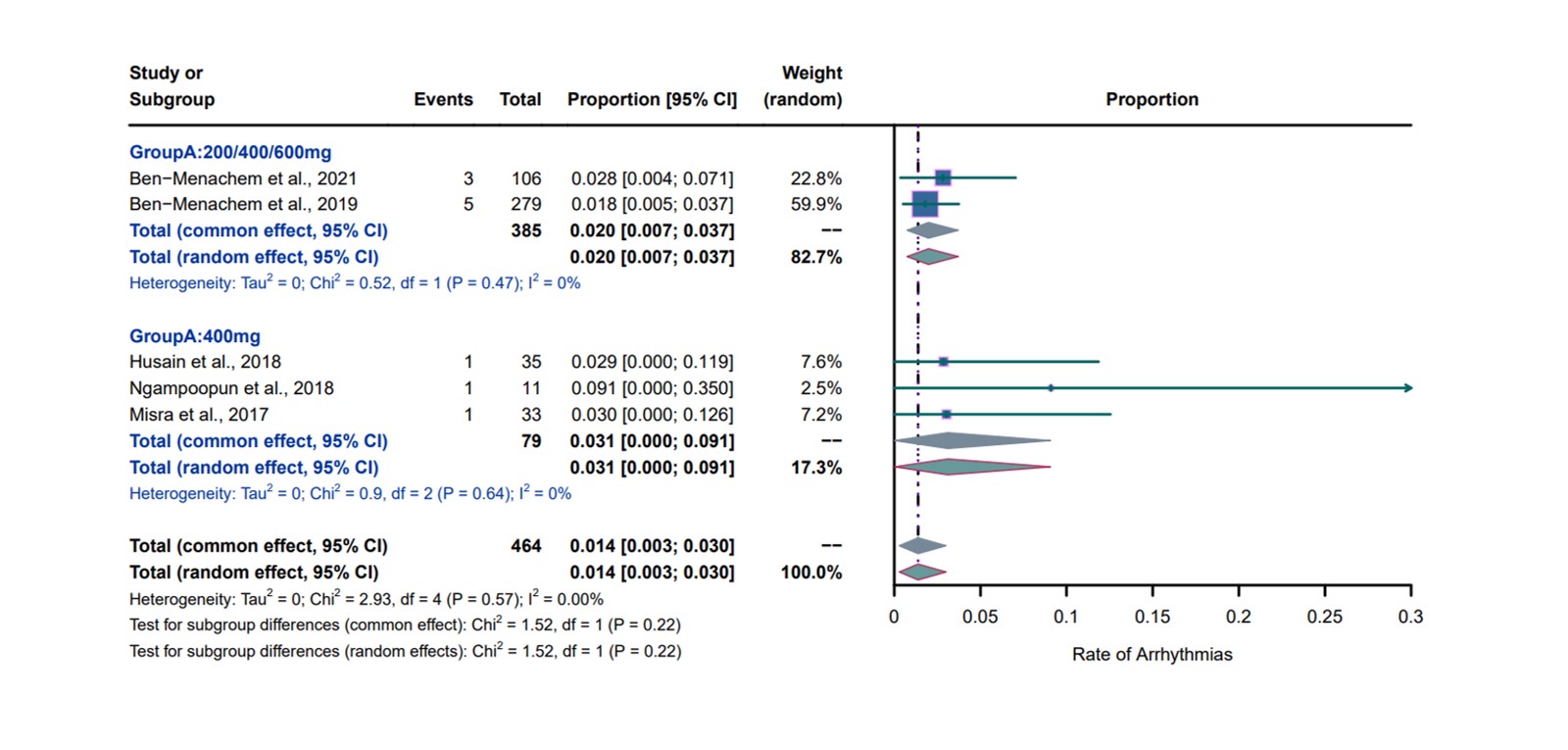
**

**Supplementary Figure 4.** Subgroup analyses were performed by dividing the LCM group into two subgroups based on the administered dose.

**
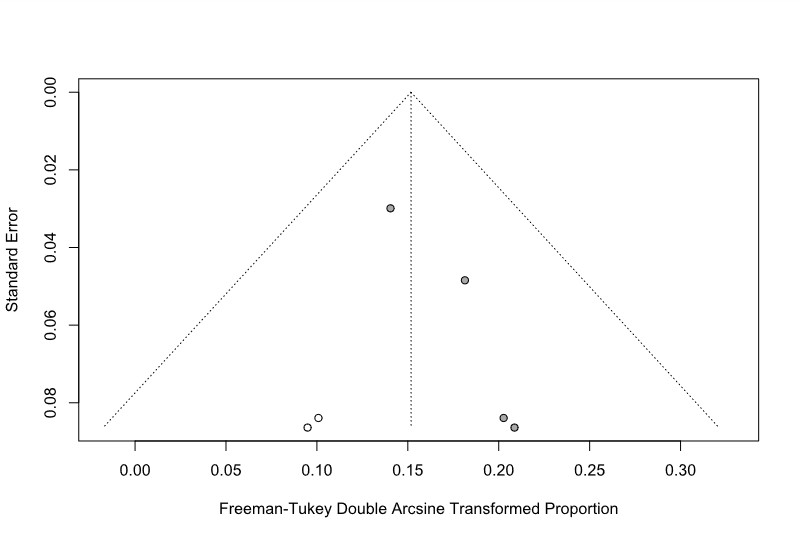
**

**Supplementary Figure 5.** The cut-and-patch method was used to test for publication bias in the LCM group
