# Supplementary material for: Risk assessment of arrhythmias related to three antiseizure medications: a systematic review and single-arm meta-analysis
Source: Front Neurol. 2024 Feb 14;15:1295368. doi: 10.3389/fneur.2024.1295368 (PMC10899418; doi:10.3389/fneur.2024.1295368)
Supplement: Supplementary file 2 [file Data_Sheet_2.DOCX]

**Search strategy：**

1. **PUBMED**
   1. **Perampanel (26):**

(("perampanel"[Supplementary Concept] OR "hydrate*"[All Fields] OR "e 2007"[All Fields] OR "e2007"[All Fields] OR "er 155055 90"[All Fields] OR "fycompa"[All Fields]) AND ("monotherapies"[All Fields] OR "monotherapy"[All Fields])) AND ("Randomized Controlled Trial" [Publication Type] OR clinical study OR clinical trial* OR clinical trial protocol OR clinical trial phase i OR clinical trial phase ii OR clinical trial phase iii OR clinical trial phase iv OR controlled clinical trial OR multicenter study OR randomized controlled Trial OR validation study OR random allocation double-blind OR single-blind OR random* OR trial* OR RCT OR Randomized Controlled Trials OR placebo)

- 1. **Levetiracetam (14):**

("Levetiracetam"[MeSH Terms] OR "Levetiracetam"[All Fields] OR "ucb l059"[All Fields] OR "ucb l059"[All Fields] OR "Keppra"[All Fields] OR "Etiracetam"[All Fields] OR "alpha ethyl 2 oxo 1 pyrrolidineacetamide"[All Fields] OR "alpha ethyl 2 oxo 1 pyrrolidineacetamide"[All Fields] OR "ucb l060"[All Fields] OR "ucb l060"[All Fields]) AND ("arrhythmias, cardiac"[MeSH Terms] OR "Cardiac Dysrhythmia"[All Fields] OR "dysrhythmia cardiac"[All Fields] OR "Cardiac Arrhythmia"[All Fields] OR "Arrhythmia"[All Fields] OR "Arrythmia"[All Fields] OR "Heart rate"[All Fields]) AND ( "Randomized Controlled Trial" [Publication Type] OR clinical study OR clinical trial* OR clinical trial protocol OR clinical trial phase i OR clinical trial phase ii OR clinical trial phase iii OR clinical trial phase iv OR controlled clinical trial OR multicenter study OR randomized controlled Trial OR validation study OR random allocation double-blind OR single-blind OR random* OR trial* OR RCT OR Randomized Controlled Trials OR placebo)

- 1. **Lacosamide (7):**

("lacosamide"[Supplementary Concept] OR "lacosamide"[All Fields] OR "lacosamide"[MeSH Terms] OR "Vimpat" [All Fields] OR "N-benzyl-2-acetamido-3-methoxypropionamide" [All Fields] OR "N benzyl 2 acetamido 3 methoxypropionamide" [All Fields] OR "N benzyl AcMeOPrNH2" [All Fields] OR "N-benzyl-AcMeOPrNH2" [All Fields]) AND ("Randomized Controlled Trial" [Publication Type] OR clinical study OR clinical trial* OR clinical trial protocol OR clinical trial phase i OR clinical trial phase ii OR clinical trial phase iii OR clinical trial phase iv OR controlled clinical trial OR multicenter study OR randomized controlled Trial OR validation study OR random allocation double-blind OR single-blind OR random* OR trial* OR RCT OR Randomized Controlled Trials OR placebo) AND ("Arrhythmias, Cardiac"[Mesh] OR " Cardiac Dysrhythmia"[All Fields] OR "Dysrhythmia, Cardiac"[All Fields] OR "Cardiac Arrhythmia"[All Fields] OR "Arrhythmia"[All Fields] OR "Arrythmia"[All Fields] OR " Heart rate "[All Fields])

1. **Web of science**
   1. **Perampanel (0):**

TS=(perampanel OR 3-(2-cyanophenyl)-5-(2-pyridyl)-1-phenyl-1,2-dihydropyridin-2-one OR perampanel hydrate* OR E-2007 OR E2007 OR ER-155055-90 OR Fycompa) AND TS=(Arrhythmias OR Cardiac Dysrhythmia OR Cardiac Arrhythmia OR Arrhythmia OR Arrythmia AND heart rate) AND TS=(controlled clinical trial OR random allocation OR double-blind OR single-blind OR placebo OR randomly OR randomized OR clinical trial* OR trial* OR rct OR random* OR Randomized Controlled Trials )

- 1. **Levetiracetam (25):**

TS=(Levetiracetam OR S-isomer Etiracetam OR Ucb L059 OR Ucb-L059 OR UCB 6474 OR UCB-6474 OR UCB6474 OR R-isomer Etiracetam OR Etiracetam, (R)- OR Keppra OR Etiracetam OR alpha-ethyl-2-oxo-1-Pyrrolidineacetamide OR alpha ethyl 2 oxo 1 Pyrrolidineacetamide OR Ucb L060 OR Ucb-L060 OR UcbL060) AND TS=(Arrhythmias OR Cardiac Dysrhythmia OR Cardiac Arrhythmia OR Arrhythmia OR Arrythmia OR heart rate) AND TS=(controlled clinical trial OR random allocation OR double-blind OR single-blind OR placebo OR randomly OR randomized OR clinical trial* OR trial* OR rct OR random* OR Randomized Controlled Trials )

- 1. **Lacosamide (10):**

TS=(Lacosamide OR Vimpat OR N benzyl 2 acetamido 3 methoxypropionamide OR N-benzyl-AcMeOPrNH2 OR N-benzyl-2-acetamido-3-methoxypropionamide OR N benzyl AcMeOPrNH2) AND TS=(Arrhythmias OR Cardiac Dysrhythmia OR Cardiac Arrhythmia OR Arrhythmia OR Arrythmia OR heart rate) AND TS=(controlled clinical trial OR random allocation OR double-blind OR single-blind OR placebo OR randomly OR randomized OR clinical trial* OR trial* OR rct OR random* OR Randomized Controlled Trials )

1. **Cochrane Library**
   1. **Perampanel (8):**

((perampanel OR E2007 OR E-2007 OR FYCOMPA OR Perampanel-D5):ti,ab,kw) AND ((MeSH descriptor: [Arrhythmias, Cardiac] explode all trees) OR (Arrhythmia OR Arrythmia OR Cardiac Arrhythmias OR Cardiac Arrhythmia OR Arrhythmia, Cardiac OR Dysrhythmia, Cardiac OR Cardiac Dysrhythmia OR heart rate))

- 1. **Levetiracetam (20):**

((MeSH descriptor: [Levetiracetam] explode all trees OR (Etiracetam, S isomer):ti,ab,kw) OR ((Etiracetam, S-isomer):ti,ab,kw OR (S-isomer Etiracetam):ti,ab,kw OR (Ucb-L059):ti,ab,kw OR (Ucb L059):ti,ab,kw OR (Keppra):ti,ab,kw OR (Etiracetam, R isomer):ti,ab,kw OR (R-isomer Etiracetam):ti,ab,kw OR (Etiracetam, R-isomer):ti,ab,kw OR (Ucb L060):ti,ab,kw OR (Ucb-L060):ti,ab,kw OR (UcbL060):ti,ab,kw OR (alpha ethyl 2 oxo 1 Pyrrolidineacetamide):ti,ab,kw OR (Etiracetam):ti,ab,kw OR (UCB6474):ti,ab,kw OR (UCB-6474):ti,ab,kw OR (UCB 6474):ti,ab,kw)) AND ((MeSH descriptor: [Arrhythmias, Cardiac] explode all trees) OR (Arrhythmia OR Arrythmia OR Cardiac Arrhythmias OR Cardiac Arrhythmia OR Arrhythmia, Cardiac OR Dysrhythmia, Cardiac OR Cardiac Dysrhythmia OR heart rate))

- 1. **Lacosamide (4):**

((MeSH descriptor: [Lacosamide] explode all trees) OR ((Vimpat OR N benzyl 2 acetamido 3 methoxypropionamide):ti,ab,kw)) AND ((MeSH descriptor: [Arrhythmias, Cardiac] explode all trees) OR (Arrhythmia OR Arrythmia OR Cardiac Arrhythmias OR Cardiac Arrhythmia OR Arrhythmia, Cardiac OR Dysrhythmia, Cardiac OR Cardiac Dysrhythmia OR heart rate))

1. **Embase**
   1. **Perampanel (46):**

('perampanel'/exp OR 'perampanel' OR '2 (1, 6 dihydro 6 oxo 1 phenyl 3, 2` bipyridin 5 yl) benzonitrile'/exp OR '2 (1, 6 dihydro 6 oxo 1 phenyl 3, 2` bipyridin 5 yl) benzonitrile' OR '2 (2 oxo 1 phenyl 5 pyridin 2 yl 1, 2 dihydro 3 pyridinyl) benzonitrile'/exp OR '2 (2 oxo 1 phenyl 5 pyridin 2 yl 1, 2 dihydro 3 pyridinyl) benzonitrile' OR '2 (2 oxo 1 phenyl 5 pyridin 2 yl 1, 2 dihydro 3 pyridyl) benzonitrile'/exp OR '2 (2 oxo 1 phenyl 5 pyridin 2 yl 1, 2 dihydro 3 pyridyl) benzonitrile' OR '2 (2 oxo 1 phenyl 5 pyridin 2 yl 1, 2 dihydropyridin 3 yl) benzonitrile'/exp OR '2 (2 oxo 1 phenyl 5 pyridin 2 yl 1, 2 dihydropyridin 3 yl) benzonitrile' OR '2 (2 oxo 1 phenyl 5 pyridin 2 ylpyridin 3 yl) benzonitrile'/exp OR '2 (2 oxo 1 phenyl 5 pyridin 2 ylpyridin 3 yl) benzonitrile' OR '2 [6` oxo 1` phenyl 1`, 6` dihydro (2, 3` bipyridin) 5` yl] benzonitrile'/exp OR '2 [6` oxo 1` phenyl 1`, 6` dihydro (2, 3` bipyridin) 5` yl] benzonitrile' OR '3 (2 cyanophenyl) 5 (2 pyridinyl) 1 phenyl 1, 2 dihydropyridin 2 one'/exp OR '3 (2 cyanophenyl) 5 (2 pyridinyl) 1 phenyl 1, 2 dihydropyridin 2 one' OR '5` (2 cyanophenyl) 1` phenyl 2, 3` bipyridinyl 6` (1`h) one'/exp OR '5` (2 cyanophenyl) 1` phenyl 2, 3` bipyridinyl 6` (1`h) one' OR 'e 2007'/exp OR 'e 2007' OR 'e2007'/exp OR 'e2007' OR 'er 155055 90'/exp OR 'er 155055 90' OR 'er 15505590'/exp OR 'er 15505590' OR 'er15505590'/exp OR 'er15505590' OR 'fycompa'/exp OR 'fycompa') AND ('heart arrhythmia'/exp OR 'heart arrhythmia' OR 'arrhythmia'/exp OR 'arrhythmia' OR 'arrhythmias, cardiac'/exp OR 'arrhythmias, cardiac' OR 'arrhytmia, heart'/exp OR 'arrhytmia, heart' OR 'cardiac arrhythmia'/exp OR 'cardiac arrhythmia' OR 'cardiac arrhythmias'/exp OR 'cardiac arrhythmias' OR 'cardiac arrythmia'/exp OR 'cardiac arrythmia' OR 'cardiac disrhythmia'/exp OR 'cardiac disrhythmia' OR 'cardiac dysrhythmia'/exp OR 'cardiac dysrhythmia' OR 'cardial arrhythmia'/exp OR 'cardial arrhythmia' OR 'ectopic heart rhythm'/exp OR 'ectopic heart rhythm' OR 'ectopic rhythm'/exp OR 'ectopic rhythm' OR 'heart aberrant conduction'/exp OR 'heart aberrant conduction' OR 'heart arrhytmia'/exp OR 'heart arrhytmia' OR 'heart arrythmia'/exp OR 'heart arrythmia' OR 'heart dysrhythmia'/exp OR 'heart dysrhythmia' OR 'heart ectopic beat'/exp OR 'heart ectopic beat' OR 'heart ectopic ventricle contraction'/exp OR 'heart ectopic ventricle contraction' OR 'heart rhythm disease'/exp OR 'heart rhythm disease' OR 'heart rhythm disorder'/exp OR 'heart rhythm disorder' OR 'heart rhythm problem'/exp OR 'heart rhythm problem' OR 'myocardial arrhythmia'/exp OR 'myocardial arrhythmia') AND ('randomized controlled trial'/de OR 'controlled clinical trial' OR 'random allocation'/de OR 'double-blind' OR 'single-blind' OR 'placebo'/de OR 'randomly' OR 'randomized' OR 'clinical trial*' OR 'trial*' OR 'rct' OR 'random*' OR 'randomized controlled trials as topic'/de)

- 1. **Levetiracetam (159):**

('levetiracetam'/exp OR 'agb 101' OR 'agb101' OR 'desitrend' OR 'e keppra' OR 'elepsia' OR 'elepsia xr' OR 'keppra' OR 'keppra xr' OR 'kopodex' OR 'l 059' OR 'l059' OR 'levesam' OR 'levetiracetam in sodium chloride' OR 'levipil' OR 'levroxa' OR 'lo 59' OR 'lo59' OR 'matever' OR 'spritam' OR 'ucb 059' OR 'ucb 22059' OR 'ucb l 059' OR 'ucb l059' OR 'ucb059' OR 'ucb22059') AND ('heart arrhythmia'/exp OR 'heart arrhythmia' OR 'arrhythmia'/exp OR 'arrhythmia' OR 'arrhythmias, cardiac'/exp OR 'arrhythmias, cardiac' OR 'arrhytmia, heart'/exp OR 'arrhytmia, heart' OR 'cardiac arrhythmia'/exp OR 'cardiac arrhythmia' OR 'cardiac arrhythmias'/exp OR 'cardiac arrhythmias' OR 'cardiac arrythmia'/exp OR 'cardiac arrythmia' OR 'cardiac disrhythmia'/exp OR 'cardiac disrhythmia' OR 'cardiac dysrhythmia'/exp OR 'cardiac dysrhythmia' OR 'cardial arrhythmia'/exp OR 'cardial arrhythmia' OR 'ectopic heart rhythm'/exp OR 'ectopic heart rhythm' OR 'ectopic rhythm'/exp OR 'ectopic rhythm' OR 'heart aberrant conduction'/exp OR 'heart aberrant conduction' OR 'heart arrhytmia'/exp OR 'heart arrhytmia' OR 'heart arrythmia'/exp OR 'heart arrythmia' OR 'heart dysrhythmia'/exp OR 'heart dysrhythmia' OR 'heart ectopic beat'/exp OR 'heart ectopic beat' OR 'heart ectopic ventricle contraction'/exp OR 'heart ectopic ventricle contraction' OR 'heart rhythm disease'/exp OR 'heart rhythm disease' OR 'heart rhythm disorder'/exp OR 'heart rhythm disorder' OR 'heart rhythm problem'/exp OR 'heart rhythm problem' OR 'myocardial arrhythmia'/exp OR 'myocardial arrhythmia') AND ('randomized controlled trial'/de OR 'controlled clinical trial' OR 'random allocation'/de OR 'double-blind' OR 'single-blind' OR 'placebo'/de OR 'randomly' OR 'randomized' OR 'clinical trial*' OR 'trial*' OR 'rct' OR 'random*' OR 'randomized controlled trials as topic'/de)

- 1. **Lacosamide (176):**

('lacosamide'/exp OR '2 (acetamido) 3 methoxy n (phenylmethyl) propanamide' OR '2 (acetylamino) 3 methoxy n (phenylmethyl) propanamide' OR '2 acetamido n benzyl 3 methoxypropionamide' OR '2 acetylamino n benzyl 3 methoxypropanamide' OR 'add 234037' OR 'add234037' OR 'ads 4101' OR 'ads4101' OR 'arkvimma' OR 'cosim' OR 'eplaid' OR 'erlosamide' OR 'harkoseride' OR 'kanilad' OR 'lackepila' OR 'lacoala' OR 'lacopat' OR 'lacosabil' OR 'lacosadel' OR 'lacosamide pain' OR 'laprysta' OR 'lendenuz' OR 'losmorid' OR 'lydraso' OR 'midza' OR 'n acetyl o methyl dextro serine benzylamide' OR 'seizpat' OR 'spm 927' OR 'spm 929' OR 'spm927' OR 'spm929' OR 'stutan' OR 'trelema' OR 'vilepsia' OR 'vimpat' OR 'vimpato' OR 'zilibra') AND ('heart arrhythmia'/exp OR 'heart arrhythmia' OR 'arrhythmia'/exp OR 'arrhythmia' OR 'arrhythmias, cardiac'/exp OR 'arrhythmias, cardiac' OR 'arrhytmia, heart'/exp OR 'arrhytmia, heart' OR 'cardiac arrhythmia'/exp OR 'cardiac arrhythmia' OR 'cardiac arrhythmias'/exp OR 'cardiac arrhythmias' OR 'cardiac arrythmia'/exp OR 'cardiac arrythmia' OR 'cardiac disrhythmia'/exp OR 'cardiac disrhythmia' OR 'cardiac dysrhythmia'/exp OR 'cardiac dysrhythmia' OR 'cardial arrhythmia'/exp OR 'cardial arrhythmia' OR 'ectopic heart rhythm'/exp OR 'ectopic heart rhythm' OR 'ectopic rhythm'/exp OR 'ectopic rhythm' OR 'heart aberrant conduction'/exp OR 'heart aberrant conduction' OR 'heart arrhytmia'/exp OR 'heart arrhytmia' OR 'heart arrythmia'/exp OR 'heart arrythmia' OR 'heart dysrhythmia'/exp OR 'heart dysrhythmia' OR 'heart ectopic beat'/exp OR 'heart ectopic beat' OR 'heart ectopic ventricle contraction'/exp OR 'heart ectopic ventricle contraction' OR 'heart rhythm disease'/exp OR 'heart rhythm disease' OR 'heart rhythm disorder'/exp OR 'heart rhythm disorder' OR 'heart rhythm problem'/exp OR 'heart rhythm problem' OR 'myocardial arrhythmia'/exp OR 'myocardial arrhythmia') AND ('randomized controlled trial'/de OR 'controlled clinical trial' OR 'random allocation'/de OR 'double-blind' OR 'single-blind' OR 'placebo'/de OR 'randomly' OR 'randomized' OR 'clinical trial*' OR 'trial*' OR 'rct' OR 'random*' OR 'randomized controlled trials as topic'/de)
